# Characterization of telomeric repeats in metaphase chromosomes and interphase nuclei of Syrian Hamster Fibroblasts

**DOI:** 10.1186/1755-8166-5-37

**Published:** 2012-09-03

**Authors:** Liudmila V Solovjeva, Sergey Ju Demin, Nadezhda M Pleskach, Maria O Kuznetsova, Maria P Svetlova

**Affiliations:** 1 Institute of Cytology RAS, 4 Tikhoretsky ave, Saint-Petersburg, 194064, Russia

**Keywords:** Chromosome, Telomeric repeats, Interstitial telomeric sequences, Telomeric associations, PNA FISH

## Abstract

**Background:**

Rodents have been reported to contain large arrays of interstitial telomeric sequences (TTAGGG)n (ITS) located in pericentromeric heterochromatin. The relative sizes of telomeric sequences at the ends of chromosomes (TS) and ITS in Syrian hamster (*Mesocricetus auratus*) cells have not been evaluated yet, as well as their structural organization in interphase nuclei.

**Results:**

FISH signal distribution analysis was performed on DAPI-banded metaphase chromosomes of Syrian hamster fibroblasts, and relative lengths of telomere signals were estimated. Besides well-distinguished FISH signals from ITS located on chromosomes ##2, 4, 14, 20 and X that we reported earlier, low-intensity FISH signals were visualized with different frequency of detection on all other metacentric chromosomes excluding chromosome #21. The analysis of 3D-distribution of TS in interphase nuclei demonstrated that some TS foci formed clearly distinguished associations (2–3 foci in a cluster) in the nuclei of cells subjected to FISH or transfected with the plasmid expressing telomeric protein TRF1 fused with GFP. In G0 and G1/early S-phase, the average total number of GFP-TRF1 foci per nucleus was less than that of PNA FISH foci in the corresponding cell cycle phases suggesting that TRF1 overexpression might contribute to the fusion of neighboring telomeres. The mean total number of GFP-TRF1 and FISH foci per nucleus was increased during the transition from G0 to G1/early S-phase that might be the consequence of duplication of some TS.

**Conclusions:**

The relative lengths of TS in Syrian hamster cells were found to be moderately variable. All but one metacentric chromosomes contain ITS in pericentromeric heterochromatin indicating that significant rearrangements of ancestral genome occurred in evolution. Visualization of GFP-TRF1 fibrils that formed bridges between distinct telomeric foci allowed suggesting that telomere associations observed in interphase cells are reversible. The data obtained in the study provide the further insight in the structure and dynamics of telomeric sequences in somatic mammalian cells.

## Background

Telomeres at the ends of chromosomes are composed of (TTAGGG)n repeats that are organized in complex chromatin structures by a number of specific binding proteins that prevent the fusion of chromosome ends and preclude recognition of the terminal parts of chromosomes as double-strand breaks. The length of telomeric sequences at the ends of chromosomes (TS) in germline stem cells is maintained constant due to specific telomere replication enzyme, telomerase, which elongates the ends of telomeres [1,2]. Some cancer cell lines also have a stable telomere length [3]. Somatic cells possess a limited concentration of telomerase, and in each round of replication the length of telomeric sequences decreases. The shortening of telomeres in somatic cells is associated with cell aging and is organ- and gender-specific [4,5]. The mean length of TS varies in different species [6]. Telomeres in humans are of the order of 5–20kb long [7]. There is quite limited data available regarding telomere length in animals. For example, TS in rat are 20–100kb [8], in ungulates 7–23kb, in dogs 12–23kb [9] and in mouse inbred strains 5–50kb [10] long. The average length of Chinese hamster telomeres is 38kb, and it is comparable with the length of inbred mouse telomeres, but it is much larger than that of human telomeres [11]. At present, there are no available data on the length of individual telomeres in Syrian hamster.

In humans and many other vertebrates, telomeric repeats are located not only at the ends of chromosomes, but are also represented in intrachromosomal domains. The size and localization of interstitial telomeric sequences (ITS) within chromosome bodies vary considerably among orders and species. Human genome contains about 50 ITS [12]. More than 50 vertebrate species analyzed (including representatives of classes *Mammalia, Reptilia* and *Amphibia*) contained at least one non-telomeric site of (TTAGGG)n sequence [13]. The analysis of human genome allowed to divide ITS into 3 classes: short ITS, consisted from a few number of tandemly oriented TTAGGG repeats, subtelomeric ITS, composed of large arrays of telomeric repeats, and fusion ITS, in which the arrays of repeats are oriented in a head-to-head fashion [12]. Short ITS are represented only by canonical telomeric repeats and are subdivided, in their turn, into 5 classes according to the analysis of flanking sequences. Short ITS were found to be flanked by interspersed repetitive elements (short interspersed nucleotide elements, SINE; long interspersed nucleotide elements, LINE; long terminal repeats, LTR), direct repetitive sequences of a few tens of base pairs, or unique sequences. Subtelomeric ITS, located not far from the chromosome ends, are composed of several hundred base pairs of canonical telomeric repeats or repeats containing base substitutions. Only two examples of fusion ITS in humans are known at present. ITS, that belong to this class, are located in the chromosome regions1q41 and 2q13 [12,14]. It has been shown that the fourth class of ITS is formed by long arrays of telomeric repeats located in pericentromeric chromosome regions of different animal species. ITS of this type were found in African and Indian elephants, marsupials, moles, fruit bats, hamsters and many other vertebrate species [13].

It was hypothesized long ago that ITS were derived from ancestral telomere fusion events during karyotype evolution [15]. However, the events of fusion of telomeric sequences are registered rarely [12], and it was proposed that different mechanisms are responsible for formation of ITS of different classes. The sequences of several human genomic loci containing short ITS were compared with orthologous sequences in 12 primate species [16]. The results of this analysis allowed to consider that short ITS appeared as a result of DNA double-strand break induction in ancestral genomic loci. A fragment of telomeric sequence could be inserted by chance at the site of the break during its repair, or, alternatively, 3’-end of the break could be used by telomerase as a primer for the synthesis of telomeric hexamers.

The four-step mechanism was proposed for organization of ITS in pericentromeric chromosome regions: fusion events, amplification or degeneration of telomeric repeats, subsequent reorganizations and breakage/fission [17]. The first step is characteristic for the species whose evolution included Robertsonian fusion events. The second one could occur during unequal crossing-over or DNA polymerase slippage. Subsequent genome reorganizations include inversions and translocations that could lead to the appearance of a new ITS in other genome domains. And, finally, the breakage/fission events may be associated with the formation of new acrocentric chromosomes.

The molecular organization of ITS was most extensively studied in the genome of Chinese hamster [12,18-20].

Not so much is known at present about distribution of ITS in the Syrian hamster. Earlier, high-resolution DAPI-banding of Syrian hamster metaphase chromosomes combined with FISH allowed us to map large pericentromeric ITS to 4 pairs of autosomes (chromosome numbers: 2, 4 14, 20) and X-chromosome [21]. The goal of this study was to determine the distribution of ITS in all other chromosomes of Syrian hamster, estimate the relative lengths of all TS and ITS, and analyze their spatial organization in interphase nuclei.

## Results

### Relative lengths of TS and ITS estimated by Q-FISH on metaphase chromosome spreads of Syrian hamster fibroblasts

Using the nomenclature of individual chromosomes reported by Li and co-workers [22], we determined the chromosome numbers of all chromosomes in 22 chromosome spreads after DAPI-staining combined with Q-FISH with PNA telomeric probe. DAPI-banding structure was highly similar to ideograms of G-banded chromosomes described by Li et al. [22]. In our metaphase chromosome preparations, the values of relative intensity of FISH signals from TS in sister chromatids of all chromosomes correlated significantly (correlation coefficient was equal to 0.71) which was in agreement with the data obtained earlier for human and mouse cells [10,23].

To obtain the mean relative TS length for each chromosome arm, we averaged relative intensities of TS FISH signals on p- or q- arms of both chromatids of homologous chromosomes in 22 metaphase spreads (Table 1). The relative intensities of FISH signals from TS of individual chromosomes of the same chromosome number were found moderately variable (Table 1). The intensity of FISH signal could be potentially reliant on the level of accessibility of FISH probe to chromosomes with variable degree of chromosome condensation during colchicine treatment of asynchronous culture. However, it has been shown that the higher degree of chromosome condensation during prolonged colcemid treatment does not lead to the decrease in FISH signal intensity [24] thus indicating that variations between FISH signal intensities of telomeres of individual chromosomes are possibly caused by other factors influencing PNA probe hybridization process. 

**Table 1:** Relative lengths of TS and ITS calculated from fluorescence intensity of FISH signals on metaphase plates

**Position on chromosome**	**Mean relative TS or ITS length**
1p	0.65+/−0.10
1q	0.46+/−0.09
2p	0.88+/−0.14
2q	0.39+/−0.07
3p	0.58+/−0.09
3q	0.41+/−0.07
4p	0.56+/−0.08
4q	0.35+/−0.06
5p	0.43+/−0.06
5q	0.40+/−0.06
6p	0.40+/−0.08
6q	0.41+/−0.06
7p	0.65+/−0.11
7q	0.37+/−0.05
8p	0.59+/−0.10
8q	0.37+/−0.08
9p	0.46+/−0.08
9q	0.38+/−0.07
10p	0.40+/−0.08
10q	0.40+/−0.08
11p	0.63+/−0.12
11q	0.39+/−0.07
12p	0.60+/−0.14
12q	0.41+/−0.07
13p	0.50+/−0.09
13q	0.39+/−0.07
14p	0.53+/−0.08
14q	0.42+/−0.06
15p	0.45+/−0.07
15q	0.38+/−0.06
16p	0.33+/−0.06
16q	0.41+/−0.07
17p	0.41+/−0.09
17q	0.38+/−0.06
18p	0.36+/−0.09
18q	0.39+/−0.06
19p	0.31+/−0.07
19q	0.41+/−0.08
20p	0.34+/−0.05
20q	0.37+/−0.07
21p	0.54+/−0.08
21q	0.45+/−0.08
Xp	0.41+/−0.09
Xq	0.62+/−0.12
Yp	0.53+/−0.14
Yq	0.33+/−0.09
2int	3.00+/−0.45
4int	0.94+/−0.19
14int	1.38+/−0.20
20int	0.44+/−0.09
Xint	2.10+/−0.50

For each metaphase chromosome spread, fluorescence intensities of FISH signals were normalized to the summarized fluorescence intensity of all signals (taken as 100%) on the spread. For each TS (1p – Yq) or ITS (2int – Xint), the mean (+/−3 SE) relative intensity of the signal for 22 chromosome spreads is presented.

The longest telomeres in Syrian hamster cells were localized in p-arms of chromosomes #1, #2 and #7, the shortest telomeres were localized in p-arms of chromosomes #16 and #19 and q-arm of the Y chromosome (Table 1). The ratio (R) of maximal to minimal mean TS FISH signal intensity for Syrian hamster chromosomes (data taken from Table 1) was found to be equal to 2.84.

No relationship between the relative telomere length and the corresponding telomere disposition in p- or q-arms was observed (Table 1). In acrocentric chromosomes, p-arm telomeres of chromosomes ##16 and 19 were slightly shorter than q-arm telomeres, and p-arm telomeres of chromosomes #17 and #18 were close in size to q-arm telomeres. In most of non-acrocentric chromosomes (##1, 2, 3, 4, 7, 8, 9, 11, 12, 13, 14, 15, 21 and Y), p-arm telomeres were longer than q-arm telomeres, p- and q-arm telomeres were almost equal in chromosomes ##5, 6, 10, 20, and only p-arm telomeres of the X chromosome were shorter than their q-arm counterparts.

All ITS were located in pericentromeric regions of chromosomes. Well distinguished FISH signals from ITS located on chromosomes ##2, 4, 14, 20 and X were observed in every metaphase cell. The relative intensity values of ITS belonging to these chromosomes are shown in Table 1. The longest and the shortest ITS among these chromosomes were located in chromosomes #2 and #20 respectively. The sensitivity of FISH with PNA probe is known to be increased over FISH with conventional DNA probes; however, it is also limited by the size of the target. Faint FISH signals from ITS represented by a small number of repeats were observed with different frequency of detection on other metacentric chromosomes except #21 (Table 2). The intensity of weak pericentromeric signals was not measured, because these signals were close to the resolution limit of the microscope. High frequency of detection of short ITS was found for chromosomes #5 and #10 (Table 2).

**Table 2:** Estimation of ITS FISH signal detection frequency in Syrian hamster chromosomes

**Chromosome number**	ITS FISH signal detection frequency *
1	15.22
2	100
3	23.91
4	100
5	84.78
6	13.04
7	28.26
8	41.30
9	23.91
10	63.04
11	23.91
12	13.04
13	43.48
14	100
15	10.87
16	**
17	**
18	**
19	**
20	100
21	0
X	100
Y	43.48

* 23 metaphase spreads from Syrian hamster (male) cells are analyzed. For each chromosome number, "100" represents the detection of FISH signal in all chromosomes. "0" means the absence of the signal in all chromosomes of the corresponding number.

** Frequency of detection of ITS in acrocentric chromosomes ##16, 17, 18, 19 is not presented in the table (see explanations in the text).

Pericentromeric FISH signals on acrocentric chromosomes could also be detected in some of chromosome spreads. FISH signals at the ends of all types of chromosomes were often seen as numerous spots resulting from the looping of telomeric sequences due to hypotonic treatment during metaphase chromosome preparation. It cannot be excluded that pericentromeric FISH signals on acrocentric chromosomes detected with low frequency represented the signals of loops containing p-arm telomeric sequences that overlapped pericentromeric regions. For this reason, the frequency of ITS detection for acrocentric chromosomes is not shown in Table 2.

### Visualization of associations of telomeric repeats in the nuclei of Syrian hamster cells

We expressed GFP-TRF1 protein in Syrian hamster fibroblasts obtained from a newborn animal to mark the location of TS and ITS. The GFP-TRF1 foci of different size representing TS and ITS are distributed in the nuclei of Syrian hamster cells without a preferential shift to the periphery or to the central part of the nuclei.

**Figure 1:**
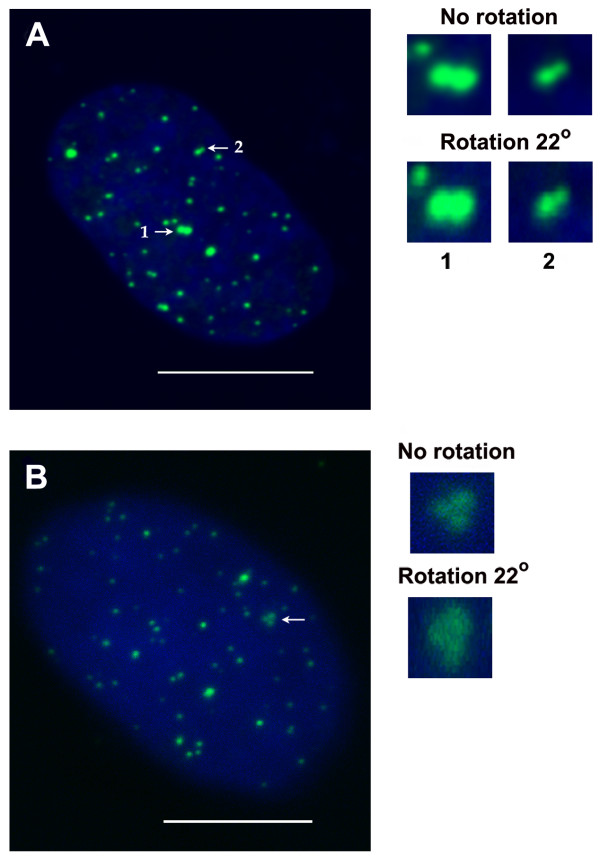
**Visualization of GFP-TRF1 and PNA FISH foci in Syrian hamster interphase nuclei.** Confocal images were captured using the Leica TCS SP5 system. Maximal projections of series of confocal sections of the nuclei are shown (**A** and **B**). The nuclear DNA is counterstained with DAPI (blue). (**A**) The nucleus expressing GFP-TRF1. GFP-TRF1 foci (green) mark location of the blocks of telomeric repeats. The foci that form clusters are indicated by arrows: 1 - the cluster of two large foci; 2 – the cluster of two small foci. (**B**) The nucleus containing PNA FISH foci (green). The cluster of three small foci is indicated by the arrow. Rotation of 3D images of the nuclei 22^o^ around x-axis using 3D projection tool of LAS AF software confirmed the association of foci in clusters. Magnified images of clusters (without rotation and rotated around x-axis) are shown on the right. Bars in A and B 10μm.

In the majority of interphase cells, some GFP-TRF1 or FISH foci were located very close to each other or juxtaposed in such a way that their fluorescence signals could be seen as foci of irregular shape. Visually well-distinguished clusters consisted mostly of 2–3 foci. These associations were registered 20% more frequently in cells subjected to FISH than in cells transfected with the plasmid expressing GFP-TRF1. The nuclei of typical cells containing foci associations, one expressing GFP-TRF1 and another subjected to FISH, are shown in Figure 1. The associations were mostly seen between the small foci. Less frequently, associations were formed between large foci and the smaller ones, and associations between the large foci could be detected relatively seldom. For example, in the nucleus shown in Figure 1A, two large foci and two small GFP-TRF1 foci associated in clusters are presented. The presence of two or several foci located very close to each other or seen as a focus of irregular shape can be easily resolved by eye in maximal projections of confocal sections of the nuclei. To confirm the formation of real clusters and exclude the visual superposition of FISH or GFP-TRF1 signals in maximal projections, we carefully examined the images of sequential confocal sections of the nuclei. We suggest that, besides well-distinguished associations, some TS foci in Syrian hamster interphase nuclei could be fused so tightly that it would be impossible to distinguish separate foci put together.

For all experiments, we used asynchronous culture of fibroblasts (early passages, i.e. the passage number 1 or 2) represented by cells in G0, G1, S and G2 stages of the cell cycle. The counting of the total numbers of GFP-TRF1 and FISH foci in randomly selected nuclei has shown that these numbers differed significantly, and FISH foci are more numerous in comparison with transfected cells containing GFP-TRF1 foci.

### Comparison of the numbers of FISH and GFP-TRF1 foci in G0- and G1/early S-phase cells

**Figure 2:**
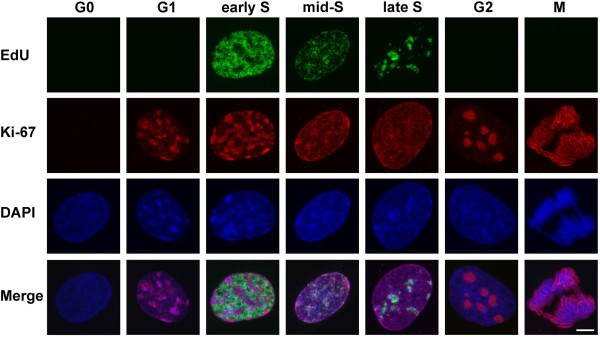
**Ki-67 and EdU staining patterns of Syrian hamster nuclei at different phases of the cell cycle.** RGB images (shown as Merge) of the maximal projections of the confocal sections of the nuclei are split into green, red and blue channels that reflect Ki-67, EdU and DAPI staining. The phases of the cell cycle are indicated. Bar 5μm.

To compare the numbers of FISH signals and GFP-TRF1 foci per cell in certain phases of the cell cycle, additional Ki-67 staining was performed. Ki-67 localization in the nuclei is cell cycle dependent [25] and linked to rRNA transcription machinery [26]. S-phase cells were marked also by 5-ethynyl-2’-deoxyuridine (EdU) incorporation. In Syrian hamster fibroblasts, we observed the patterns of Ki-67 nuclear distribution resembling those described earlier for human fibroblasts and ES cells [27-29], but with minor distinctive features. Quiescent fibroblasts and presumably early G1 cells exhibited very faint dot-like Ki-67 nucleoplasm staining. EdU-negative late G1 cells were characterized by low level of nucleoplasm staining and bright perinucleolar staining around multiple nucleoli. Ki-67 staining patterns of EdU-positive S-phase Syrian hamster cells differed from those described for mammalian cells earlier [28,29]. Early S-phase pattern was very similar to late G1 one. In mid and late S-phase, loose nucleoli were not well distinguished on intense nuclear background. In G2 phase, the intensity of nuclear background staining was reduced and accompanied with the appearance of well-defined and bright nucleoli (Figure 2). The number of brightly Ki-67-stained nucleoli in G2 cells was usually 5–7, i.e. it was higher than that in human fibroblasts and ES cells [27,29]. 

**Figure 3:**
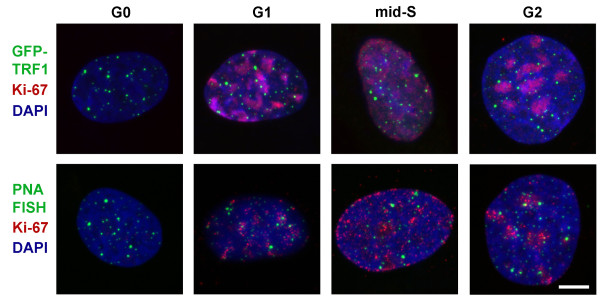
**Visualization of GFP-TRF1 and FISH foci in Syrian hamster nuclei after immunostaining with antibodies to Ki-67 proliferative marker.** Maximal projections of confocal sections are shown. The phases of the cell cycle analysed are indicated. Bar 5μm.

The number of telomeric foci was estimated in cells progressing through the cell cycle (Figure 3). The majority of cells in asynchronous Syrian hamster cell culture were in G0 and G1 phases of the cell cycle that was in accordance with flow cytometry data obtained for cycling mammalian fibroblasts [30]. FISH and GFP-TRF1 foci were counted in Ki-67-negative G0 cells and G1/early S-phase cells. Ki-67 staining pattern in late G1 was poorly distinguished from that in early S-phase, that is why the counting of foci specifically in G1 was impossible. 

**Figure 4:**
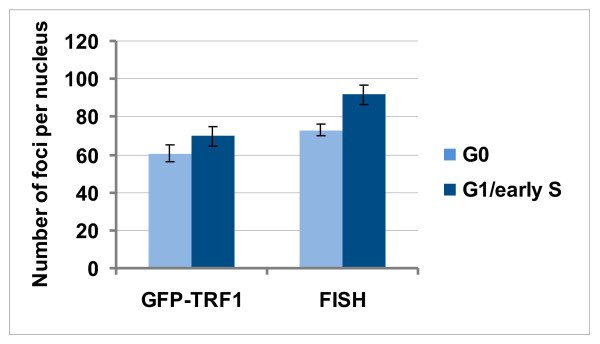
**The mean total numbers of GFP-TRF1 and FISH foci in G0 and G1/early S-phase cells of Syrian hamster.** The column chart was created using Microsoft Office Excel 2007. 32–39 cells for each column were analysed. Error bars represent 3 standard errors.

The mean total numbers of GFP-TRF1 and FISH foci per nucleus are increased in G1/early S-phase in comparison with G0 (Figure 4). It was found that yeast telomeres replicate only in late S-phase, and human telomeres replicate throughout the duration of S-phase [31,32]. There is no data on timing of telomere replication in Syrian hamster, and it cannot be excluded that duplication of some telomeres could occur in early S-phase thus increasing the number of GFP-TRF1 and FISH foci at this stage of the cell cycle. 

The mean total numbers of GFP-TRF1 foci per nucleus in G0 and G1/early S-phase were less than those of FISH foci in the corresponding phases (Figure 4). This observation could be interpreted in two ways. Some small GFP-TRF1 foci may be simply unseen due to the limit of GFP brightness, otherwise expression of GFP-TRF1 could lead to the increase in formation of TS fusions registered as single foci.

### Mobility of GFP-TRF1 foci in the nuclei of living fibroblasts

**Figure 5:**
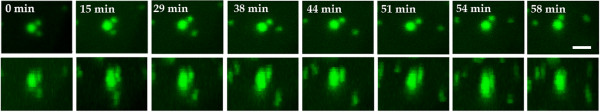
**Live imaging of GFP-TRF1 foci in the nucleus of the Syrian hamster fibroblast.** In living cells expressing GFP-TRF1, telomeric foci move stochastically. During 1.5h of observation, no fusion of the represented foci was observed. The upper line represents xy-projection of a fragment of the nucleus, and the bottom line – 45^o^ rotation of the same fragment around x-axis. Bar 2μm.

We were interested to see whether telomere clustering events could be noticed *in vivo* in living Syrian hamster cells. In transiently transfected cells, a constrained stochastic movement of chromosome ends tagged with GFP-TRF1 was observed. Figure 5 shows an example of relocation of two small foci relative to a bigger one in the nucleus of a Syrian hamster fibroblast. The distance between the foci changed during the time of observation (1.5h), but a complete association of the foci was not recorded. We suggest therefore that the formation of “newborn” associations of telomeric repeats in interphase cells is a relatively rare event. Dynamic telomere associations were studied earlier in U2OS and transformed ECV304 human cell lines [33,34]. In contrast to our observations on primary Syrian hamster fibroblasts, telomere collisions and temporal dissociations of telomere clusters were recorded at times by these authors suggesting a faster telomere dynamics in transformed and cancer cells. 

### Visualization of GFP-TRF1-tagged telomere-telomere bridges

**Figure 6:**
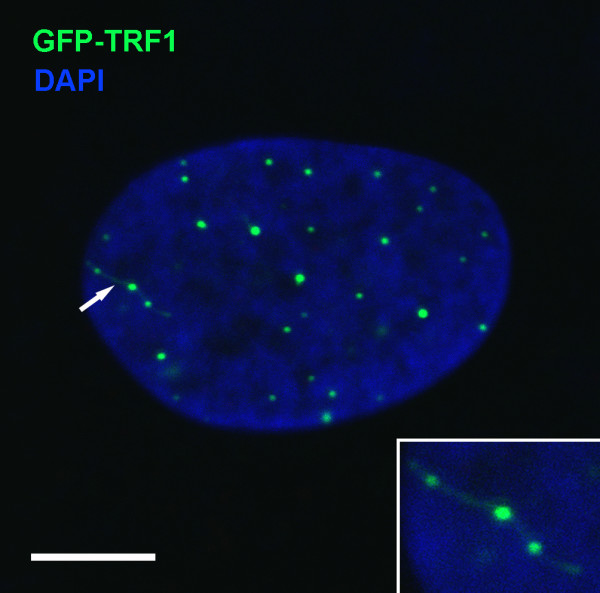
**Visualization of bridges between GFP-TRF1 foci.** The interphase nucleus of the fibroblast at passage 2 is shown. A few foci connected by a bridge are indicated by the arrow. Bar 5μm. In the bottom right corner, the magnified image of the bridge between the foci indicated by the arrow is presented.

18% of fixed cells after transfection with GFP-TRF1 plasmid (66 cells analyzed) contained green bridges that connect two or more foci located close to each other (Figure 6). These bridges possibly result from spatial separation of TS clusters into individual TS. The occasional displacement of chromosome territories in interphase cells could produce disjunctions of fused telomeric ends resulting in the appearance of telomere-telomere filamentous bridges associated with GFP-TRF1. The presence of DNA within green bridges was indistinguishable in blue channel images due to dense DAPI background.

## Discussion

The average Syrian hamster telomere length determined by Southern blot analysis of telomere restriction fragments was 19kb [35]. Using Q-FISH with telomeric PNA probe, we were the first to evaluate the relative lengths of all telomeres of individual Syrian hamster chromosomes. Q-FISH with PNA telomeric probe is an advantageous approach for the quantitative measurement of the length of DNA fragments hybridized with the probe. The resolution of Q-FISH was estimated to be 200 bp [36], and the mean fluorescence intensity of telomeres measured by Q-FISH correlated with the mean size of telomere restriction fragments [23].

The R value (the ratio of maximal to minimal mean relative TS length values) for human chromosomes obtained by Q-FISH telomere analysis was 2.1 [37]. The Syrian hamster R value measured in this study was estimated to be 2.84. For comparison, one rodent species, the Iberian shrew, has extremely long telomeres on the short arms of acrocentric chromosomes containing 213kb on average (maximally 300kb) and very short telomeres on the other chromosomal ends containing 3.8kb on average (R is about 56) [38].

A strong positive correlation between the telomere length and the size of corresponding chromosome arm or the length of the entire chromosome was observed in human cells [37,39]. It has been suggested previously that telomere length could be affected by centromere position and is regulated proportionally to the distance between telomere and centromere [40]. Telomere length measurements after Q-FISH on mouse acrocentric chromosomes have revealed that p-arm telomeres are shorter than their q-arm counterparts [10]. In all Chinese hamster non-acrocentric chromosomes analyzed, p-arm telomeres are shorter than q-arm telomeres [40]. As we have shown in this study, in the majority of Syrian hamster non-acrocentric chromosomes (in 14 out of 19) p-arm telomeres do not fit this tendency and are longer than q-arm telomeres.

Pericentromeric location of ITS in some Syrian hamster chromosomes was observed previously by several groups of researchers [13,41,42]. The relative sizes of all ITS in Syrian hamster species were measured in the present study. FISH signals in the pericentromeric regions of chromosomes 2, 4, 14, 20 and X were observed in every metaphase cell, and the largest signal was located on chromosome #2. All other non-acrocentric chromosomes except #21 also contained short ITS in pericentromeric regions that were detected with a lower frequency.

The similar results were obtained earlier on Chinese hamster primary fibroblast cell line [43]. Pericentromeric regions of all Chinese hamster chromosomes except chromosome #1 and Y hybridized with telomeric probe. Using chromosome-specific painting probes and G-banding, the comparative maps between 20 rodent species have been established [44,45]. On the base of karyotype homology analysis, the map of ancestral Muroidea karyotype was proposed [45,46]. 24 fusion/breakage events occurred during evolution of *Mesocricetus auratus* karyotype [45]. The fine sequence analysis of the species is needed to evaluate if some of fusions resulted in the appearance of ITS in pericentromeric regions. Q-arms of metacentric chromosomes #1 and #12 of *Mesocricetus auratus* have the high level of homology with the entire ancestral chromosomes #3 and #7 allowing to hypothesize that these chromosomes might originate from Robertsonian fusions of ancestral chromosomes with unidentified yet chromosomal segments, and retain telomeric sequences in the newly formed centromeres. It is more likely that pericentromeric ITS arise in karyotype evolution by different mechanisms including intra-chromosomal translocations and, probably, whole arm pericentic inversions. It is possible that subsequent amplification of telomeric sequences retained in the pericentromeric regions could occur resulting in the appearance of large (TTAGGG)n blocks that could be detected in some of chromosomes. 10 of 11 fragile chromosome sites are localized within pericentromeric bands containing ITS in Chinese hamster [43,47] indicating that pericentromeric ITS could be a subject of further rearrangement events in evolution.

It has been shown that formation of telomeric aggregates is a regular feature of tumor cells [48]. Fusions of telomeres characterize cells overexpressing c-Myc, a protein that contributes to genomic instability [49]. Some examples of telomere interaction in normal vertebrate cells were reported. Telomere dimers result from specific interactions between the two ends of each chromosome in human sperm cells [50]. Telomere ends of some chromosomes in Chinese hamster embryonic cells were preferentially organized in clusters [51]. Telomeres of NOR-containing acrocentric chromosomes in human lymphocytes usually cluster at the periphery of nucleoli [52]. Telomeric associations were observed in normal human fibroblasts, and the number of associations in non-cycling cells was increased in comparison with replicating cells [53].

Associations of FISH and GFP-TRF1 telomeric foci (mostly 2–3 foci per cluster), well-distinguished by eye, were observed in interphase Syrian hamster cells. Visualization of telomere-telomere bridges covered by GFP-TRF1 protein in fixed cells allows to suggest that formation of telomere associations could be reversible.

In G0 and G1/early S-phase Syrian hamster cells, the total number of telomeric foci per nucleus in cells expressing GFP-TRF1 was less than that in cells subjected to FISH. TRF1 dimers bind double-stranded regions of telomeric DNA through Myb domains located in C-terminal fragments of each monomer. TRF1 possesses extreme structural flexibility and could pair distinct telomeric tracts *in vitro*[54-56]. It has been shown using CHIP-Seq analysis that TRF1 and TRF2 could bind some of non-telomeric heterochromatin-like repeats in the chromatin of tumor cell line [57]. However, it is unknown whether such kind of interactions could occur *in vivo*. Stochastic movement of telomeres is a general feature of chromosomes; when neighboring telomeres come close enough to each other, GFP-TRF1 dimers could mediate their association.

The nature of telomere end-to-end associations and their functional importance remain unknown. Most possibly, they represent temporal fusions being formed by TRF1 and/or other proteins integrated in shelterin complex. Telomere clustering observed in interphase evidently occurs between telomeres located in adjacent chromosome territories and is probably dependent on individual chromosome properties like telomere length and mobility of telomere ends. Further research is needed to confirm the existence of preferential telomere-telomere interactions in Syrian hamster cells.

## Conclusions

Characterization of telomeric repeats in Syrian hamster cells was done in this study. It was found that the ratio of maximal relative length of TS to minimal one in Syrian hamster does not differ significantly from that in humans. ITS were found in pericentromeric regions of the majority of metacentric chromosomes, indicating that the extensive chromosome rearrangements that involved telomeric sequences occurred during the karyotype evolution. Some telomeres in interphase cells are juxtaposed to each other and form associations. Usually, clusters of 2–3 TS foci were observed in cells subjected to FISH or expressing GFP-TRF1. Visualization of green bridges that connect two or several telomeres in cells expressing GFP-TRF1 is probably due to separation of fused TS, thus reflecting the dynamic behavior of telomeric ends in interphase.

## Methods

### Isolation of skin fibroblasts

The primary fibroblast culture was obtained from the skin of newborn or adult (1 year old) Syrian hamster using two methodological approaches. In the first one, the so-called “migration method”, small fragments of dissected skin were placed in Petri dishes under pieces of glass microscope slides and kept during a week in MEM culture medium supplemented with 15% foetal calf serum, aminoacids and antibiotics until fibroblasts covered an area of several cm^2^, then cells were detached by treatment with Trypsin-EDTA solution and plated in Petri dishes. In the second approach, fibroblasts were isolated by enzymatic digestion of skin sections (“enzymatic method”). Pieces of dissected skin were washed twice with PBS by centrifugation for 3min at 120 x g. The pellet was resuspended in 3 volumes of enzyme mixture: 10 mg/ml collagenase IV from *Clostridium histoliticum*, 2.5 U/ml dispase, 0.0001% Trypsin-EDTA in PBS containing Ca^2+^ and Mg^2+^ and incubated for 20min at 37^o^ C with shaking. The pieces were then dissociated into separate cells by pipetting, centrifuged at 550 x g for 10min, washed twice with PBS by centrifugation at 270 x g for 5min. After that, the cells were resuspended in culture medium and placed in CO_2_-incubator. The density of plating was approximately 2-3x10^4^ cells per 1 cm^2^ of the tissue culture flask surface.

The morphology and growth rate of fibroblasts isolated by different approaches, “migration” or “enzymatic”, did not differ, and both were used for performing FISH as well as for transient transfection.

### Metaphase chromosome spread preparation

Mitotic chromosome spreads were prepared from fibroblasts at passage 3. The cells were treated with colchicine (50μg/ml final concentration) for 4–5h before harvesting. After treatment with hypotonic solution (75 mM KCl: 1% Na citrate, 1:1) for 23min at 37°C, the cells were fixed with a mixture of glacial acetic acid and methanol (1:3), dropped onto the ice-cold glass slides and air-dried. The slides were kept at −80°C until FISH was performed.

### FISH and DAPI staining of metaphase chromosomes

Slides were stored at −80°C for 2 months before FISH with FITC-labelled PNA probe (DAKO, Telomere PNA FISH Kit/FITC, Cat. No. K5325) was performed according to manufacturer’s recommendations. The slides were stained with 0.5 μg/ml DAPI (4',6-diamidino-2-phenylindole) in PBS solution for 10min, rinsed in water and covered with Citifluor antifade solution (Marivac, Canada).

### FISH on interphase nuclei

Fibroblasts were plated on glass microscope slides the day before fixation. Cell fixation and pre-treatments were done as described [58]. Microscope slides were stored in 50% formamide/2x SSC for a week before performing Q-FISH with PNA telomeric probe (DAKO) according to manufacturer’s recommendations.

### Transfection of fibroblasts, slide fixation and DAPI staining

Fibroblasts were plated on square glass coverslips the day before transfection. The temporal transfection of Syrian hamster fibroblasts was performed using Lipofectamine 2000 transfection reagent (Invitrogen). Construction of the plasmid encoding N-terminally GFP-tagged TRF1/Pin2 was described earlier [59]. The slides were fixed at 20h after transfection in 4% formaldehyde in PBS at +4°C, rinsed in PBS, kept 15min in 70% ethanol, washed in PBS, stained with 0.5 μg/ml DAPI in PBS solution for 10min, washed in water and covered with Citifluor antifade solution (Marivac, Canada).

### EdU labeling and Ki-67 staining

For EdU labeling, we used Click-It EdU Alexa Fluor 488 Imaging Kit (Invitrogen, Cat. No C10337) according to manufecturer’s recommendations. Briefly, EdU was added to growth medium (10μM final concentration) for 15min. The cells were fixed as described above, rinsed with 3% BSA solution in PBS and permeabilized with 0.5% Triton X-100 in PBS for 20min at RT, then washed with 3% BSA in PBS and treated for 30min with Click-It coctail containing Alexa Fluor 488 azide as one of the components.

After washing with 3% BSA in PBS, the cells were incubated in 1% Blocking Reagent (Roche) with 0.02% Tween 20 for 30min. Ki-67 was detected with rabbit polyclonal antibodies to Ki-67 (abcam, 1:200) and secondary goat anti- rabbit IgG antibodies conjugated with Alexa Fluor 568 (Invitrogen, 1:400). DNA was counterstained with DAPI and mounted in antifade solution as described above.

### Microscopy and image acquisition

For image acquisition, the confocal Leica TCS SP5 system equipped with 100/1.4 oil immersion objective, 488nm argon and 405nm diode lasers and Leica LAS AF software was used. Series of confocal sections were collected with the step size 0.25μm, and maximal projections of the series were obtained. The image size was 1024x1024 pixels. The gain of the signal was adjusted to obtain a linear working range.

### Time-lapse live cell imaging

The cells were seeded on Lab-Tek borosilicate coverglass-bottomed chambered slides (NUNC, Cat. No. 155383), and, 20h after transfection with GFP-TRF1 plasmid, the movement of telomeric repeats was analyzed using the confocal system described above. The microscope stage was inside the environmental chamber with temperature control set at 37°C. Z-sections (7–12 sections per nucleus) of the nuclei were collected as described above. The series of images were captured sequentially with 3min-intervals, the total duration of single cell imaging did not exceed 1.5h. 3D Projection tool in the Visualization section of Leica LAS AF software was used for creating 3D projections of the nuclei. Movies of the rotation of 3D projections were obtained using Create Movie box of this tool. For presenting z-axis information, 3D projections were rotated by 45 degrees around x-axis.

### Processing of confocal images of metaphase chromosomes with FISH signals

Images of DAPI-banded chromosomes were analyzed after processing in Adobe Photoshop 9.0 program as described previously [21]. Fluorescence intensity of FISH signals of individual TS and ITS was measured on images using ImageJ 1.43 (NIH, USA) program, and the relative fluorescence intensity of individual FISH signal was calculated as the ratio of its intensity and summarized fluorescence intensity of all signals on the metaphase chromosome spread.

### Processing of confocal images of interphase nuclei with FISH signals or GFP-TRF1 foci

The number of foci in the nuclei was quantified by direct counting on the images obtained as maximal projections of the series of confocal sections of the nuclei.

3D Projection tool in the Visualization section of Leica LAS AF software was used to create 3D projections of the nuclei for the analysis of mutual spatial arrangement of TS and ITS foci. Movies showing the rotation of 3D projections were created using Create Movie box of 3D Projection tool. For presenting z-axis information, 3D projections were rotated by 22 or 45 degrees around x-axis.

## Abbreviations

DAPI, 4',6-Diamidino-2-Phenylindole; EdU, 5-Ethynyl-2’-Deoxyuridine; FISH, Fluorescence In Situ hybridization; GFP-TRF1, TRF1 fused with Green Fluorescent Protein; ITS, Interstitial Telomeric Sequence; PNA, Peptide Nucleic Acid; Q-FISH, Quantitative Fluorescence In Situ hybridization; R, The Ratio of maximal to minimal mean relative TS length values; TRF1, Telomeric Repeat binding Factor 1; TS, Telomeric Sequence at the end of chromosome.

## Competing interests

The authors declare that they have no competing interests.

## Authors’ contributions

LVS carried out FISH experiments and immunostaining, collected confocal images, performed the measurements of the relative length of TS and ITS. SJD carried out the processing of DAPI stained chromosomes and made karyotype analysis. NMP isolated Syrian hamster fibroblasts from the skin of the newborn animals, cultivated the cells, prepared metaphase spreads. MOK performed computer data analysis. MPS designed the project, made transfection of the fibroblasts, coordinated the study and wrote the manuscript. All authors read and approved the manuscript.
